# Spontaneous γH2AX foci in human dermal fibroblasts in relation to proliferation activity and aging

**DOI:** 10.18632/aging.102067

**Published:** 2019-07-09

**Authors:** Vadim Zorin, Anna Grekhova, Margarita Pustovalova, Alla Zorina, Nadezhda Smetanina, Natalia Vorobyeva, Pavel Kopnin, Ilmira Gilmutdinova, Alexey Moskalev, Andreyan N. Osipov, Sergey Leonov

**Affiliations:** 1 Human Stem Cells Institute, Moscow 119333, Russia; 2 State Research Center - Burnasyan Federal Medical Biophysical Center of Federal Medical Biological Agency, Moscow 123098, Russia; 3 Emanuel Institute for Biochemical Physics, Russian Academy of Sciences, Moscow 119991, Russia; 4 Moscow Institute of Physics and Technology, Dolgoprudny, Moscow Region 141700, Russia; 5 Semenov Institute of Chemical Physics, Russian Academy of Sciences, Moscow 119991, Russia; 6 N.N. Blokhin National Medical Research Oncology Center, Ministry of Health of Russia, Moscow 115478, Russia; 7 FSBI “National Medical Research Center for Rehabilitation and Balneology”, Ministry of Health of Russia, Moscow 121099, Russia; 8 Laboratory of Molecular Radiobiology and Gerontology, Institute of Biology of Komi Science Center of Ural Division of Russian Academy of Sciences, Syktyvkar, Russia; 9 Laboratory of Post-Genomic Research, Engelhardt Institute of Molecular Biology, Russian Academy of Sciences, Moscow, Russia; 10 Institute of Cell Biophysics, Russian Academy of Sciences, Pushchino, Moscow Region 142290, Russia

**Keywords:** dermal fibroblasts, aging, γH2AX foci, proliferation, cellular senescence, β‐galactosidase, сlonogenic assay

## Abstract

We assessed the effects of donor age on clonogenicity, proliferative potential, and spontaneous γH2AX foci in the proliferating (Ki67 +) and senescent (SA β-gal +) cultures of skin fibroblasts isolated from 34 donors of different age (23-82 years). Here, we demonstrated that neither the colony forming effectiveness of proliferating (Ki67+) fraction of the fibroblasts nor the average number of γH2AX foci of the same fraction does not depend on the age of the donor. The correlation between the number of γH2AX foci and the donor’s age was reliable in quiescent (Ki67-) cells. The average number of γH2AX foci in quiescent fibroblasts of donors older than 68 years was about two times higher than in the same cells of up to 30 years old donors. The number of γH2AX foci demonstrated a statistically significant positive correlation with the fraction of proliferating cells in fibroblast cultures. On average, proliferating cells have twice as many the γH2AX foci in comparison with the quiescent cells. Within a population of proliferating (Ki67+) cells, the degree of senescence correlated with a relative declining of constitutive γH2AX foci number, whereas in the population of quiescent (Ki67-) cells, it was proportional to augmenting the number of the γH2AX foci. Our data on a statistically significant (p=0.001) correlation between the age of the donor and the number of constitutive γH2AX foci in quiescent cells, could point out the ongoing DNA-damage response due in the maintenance of the senescent state of cells.

## Introduction

Skin aging is a multi-factorial process that affects nearly every aspect of its biology and function [1]. However fundamental molecular mechanisms of skin aging are associated with fibroblasts, the primary dermal cell population – the main function of which is to produce and organize an extracellular matrix (ECM) that provides the skin with structural integrity and elasticity [2–4]. The young skin fibroblasts produce and adhere to ECM, consisting of mainly of type I collagen fibrils it [5]. Aging decrease the number of functionally active fibroblasts in the skin, reduce their biosynthetic activity and content of collagen, the main structural dermal component [6]. The structural alterations and degradation of ECM, thought to result in dermal thinning, increasing wrinkling and loss of elasticity [6,7]. This imbalance of ECM homeostasis further stimulates fragmentation of collagen fibrils in a self-sustaining vicious circle.

The accumulation of genetic alterations arising from the ineffective or incorrect repair of spontaneous DNA damage (attacks of metabolic free radicals produced, replication and recombination errors, spontaneous chemical changes) is one of the most significant causes of aging and oncotransformation of cells [8]. Some authors consider the accumulation of DNA damage in cells as a universal cause of age-dependent changes at cellular level [9–11]. DNA double-strand breaks (DSBs) are the most intensively investigated form of the damage among the variety of spontaneous DNA lesions. Indeed, DSBs are the most critical DNA alterations that can define the fate of cells and, if repaired incorrectly or inefficiently, can lead to serious cytogenetic abnormalities, cell death, inactivation of tumor suppressor genes and/or activation of oncogenes [12–15]. Moreover, the formation of a particular phenotype inherent to senescence linked to the functional state of repair of DSBs, as well as to the accumulation of DSBs [16].

Recently, an indirect method based on the immunocytochemical (ICC) analysis of proteins involved in the repair of DSBs became increasingly popular for studying changes in a small amount of DSBs in living cells. During DSBs DNA repair, complex dynamic microstructures, consisting of thousands of copies of proteins can be visualized by ICC staining in the form of bright dots, so-called DNA repair protein foci [17,18]. It is believed that one focus corresponds to a single or multiple DSBs repair site(s) [19]. The DNA damage response kinases of the PI3-family (Ataxia telangiectasia mutated, ATR, DNA-PKcs and especially ATM) phosphorylate core histone H2AX(γН2АХ) on serine 139 in response to the formation of DSBs. Therefore, the ICC-assisted measurement of γН2АХ foci indicates the presence of DNA double-strand breaks and DNA repair site(s) [20,21]. The constitutive level of γH2AX expression in live cells, untreated by exogenous agents, likely represents DNA damage by endogenous oxidants generated during cellular respiration [22].

We studied spontaneous (constitutive) γH2AX foci formation in human dermal fibroblasts in relation to proliferation activity and aging of the donors of the fibroblasts. To estimate the impact of proliferation state, we assessed the number of γH2AX foci in Ki67 positive (Ki67+) and Ki67 negative (Ki67-) cells. The Ki67 protein is a marker of cell proliferation. It is present in proliferating (G1, S, G2, M), but absent in quiescent (G0) cells [23]. Furthermore, we analyzed the correlations between γH2AX foci number, senescent (SA-βgal+) cell fraction and the number of colonies formed by skin fibroblasts from donors of different age.

## RESULTS

### Characteristics of donors: age, sex and clonogenicity

The study involved 34 healthy donors, 25 women, and 9 men, who were divided into 4 age groups – Young (before 30 years), Middle-aged I (from 30 till 45 years), Middle-aged II (from 46 till 59 years) and Elderly or Senior (above 68 years) (see Table 1).

**Table 1:** Characteristics of the donors: age, sex and clonogenicity (ECO-f^1^, PP^2^).

**Group**	**Рatient number**	**Аge**	**Sex**	**ECO-f**	**PP**
**Young**	1.	23	M	9,0	2,0
	2.	23	F	28,3	1,7
	3.	27	M	49,3	2,0
	4.	28	М	29,7	1,3
	5.	29	F	54,7	2,5
**Mean±SE**		**26.0±1.3**	**-**	**34.2±8.2**	**1.9±0.2**
**Middle-aged I**	6.	31	F	42,3	2,1
	7.	31	F	43,3	1,6
	8.	31	F	30,0	1,8
	9.	33	F	56,7	1,9
	10.	33	F	24,7	1,5
	11.	34	F	9,7	1,1
	12.	35	F	30,3	1,3
	13.	36	М	33,0	1,5
	14.	38	F	59,0	2,2
	15.	41	F	42,0	1,7
	16.	42	F	8,7	1,1
	17.	43	F	40,3	1,7
	18.	43	F	40,3	2,2
	19.	45	F	53,0	2,6
**Mean±SE**		**36.9±0.4**	**-**	**36.7±4.4**	**1.7±0.2**
**Middle-aged II**	20.	46	М	42,3	1,93
	21.	46	М	15,0	1,5
	22.	48	М	57,3	1,93
	23.	48	F	37,0	1,78
	24.	48	F	61,0	2,23
	25.	54	F	11,0	1,57
	26.	57	М	19,7	1,35
	27.	59	F	62,0	2,25
	28.	59	F	16,0	1,04
	29.	59	F	42,3	1,98
	30.	59	F	51,3	2,20
**Mean±SE**		**53.0±0.3**	**-**	**37.7±6.0**	**1.8±0.2**
**Senior**	31.	68	F	49,7	1,79
	32.	69	F	37,3	2,01
	33.	70	F	19,7	1,05
	34.	82	М	44,0	2,2
**Mean±SE**		**72.3±3.3**	**-**	**37.7±6.5**	**1.8±0.3**

^1^ Colony forming effectiveness of the fibroblasts. ^2^ Proliferative potential.

The ability to form colonies in vitro represents one of the "gold standard" methods for the assessment of the clonogenic survival of cells [24]. Another important characteristic of fibroblasts is their proliferation potential (PP). During the conventional skin fibroblast cultivation, the cell-precursors of fibroblasts create three types of colonies - dense, mixed and diffuse colonies [25]. The cells of dense colonies possess the highest PP, whereas the cells of diffuse colonies possess the lowest PP.

According to our data, neither the PP nor colony forming effectiveness (ECO-f) of the fibroblasts did not demonstrate a statistically significant difference between the groups of different age (Table 1).

### Сlonogenicity, proliferation activity, and percent of senescent (SA β-galactosidase positive) fibroblasts are not correlated with donor age

**Figure 1:**
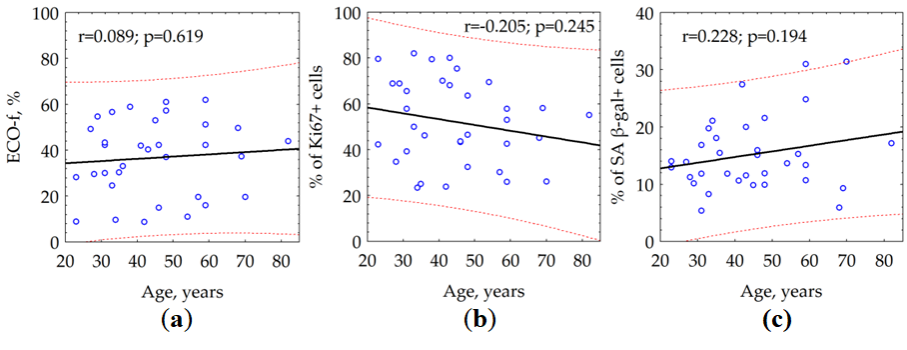
The effect of donor age on (**a**) colony formation (ECO-f), (**b**) the fraction of proliferating (Ki67 +) and (**c**) the fraction of senescent (SA β-gal +) skin fibroblasts.

To estimate the impact of donor age, we performed a correlation analysis of ECO-f, PP, the fractions of proliferating (Ki67 +) and senescent (SA β-gal +) cells. We found that neither the ECO-f (Fig. 1a) nor the proliferative potential (not presented) of skin fibroblasts does not depend on the age of the donor. Similarly, there was no statistically significant correlation between neither the fractions of proliferating (Fig. 1b) nor senescent (Fig. 1c) fibroblasts and the age of the donor. Of note, there was a statistically significant positive correlation (data not shown) between ECO-f and the fraction of proliferating cells (r = 0.458, p = 0.006) and inverse correlation between ECO-f and the proportion of senescent (SA β-gal +) cells (r= -0.492, p = 0.003).

### γH2AX foci number of quiescent cells increases with donor age

**Figure 2:**
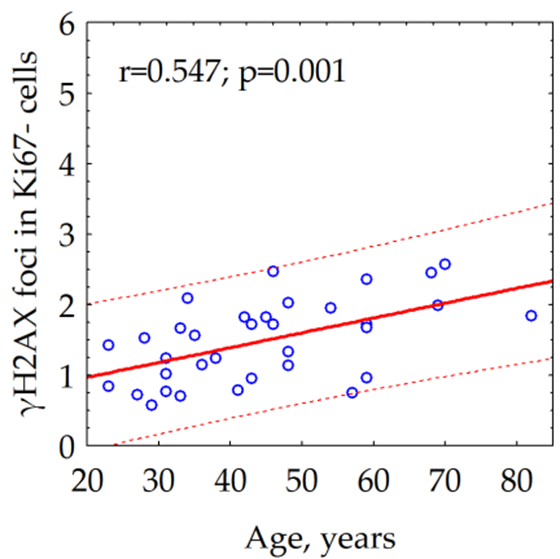
Correlation between the donor's age and the number of γH2AX foci in quiescent (Ki67-) skin fibroblasts.

We observed a statistically significant (p = 0.001) correlation between the age of the donor and the number of the γH2AX foci in quiescent cells (Fig. 2). Notably, we could not reveal the relationship between the age of the donor and the average number of γH2AX foci (data not shown) within both total cell population (r = -0.095, p = 0.593) and fraction of proliferating (Ki67 +) cells (r = -0.241, p = 0.169).

**Figure 3:**
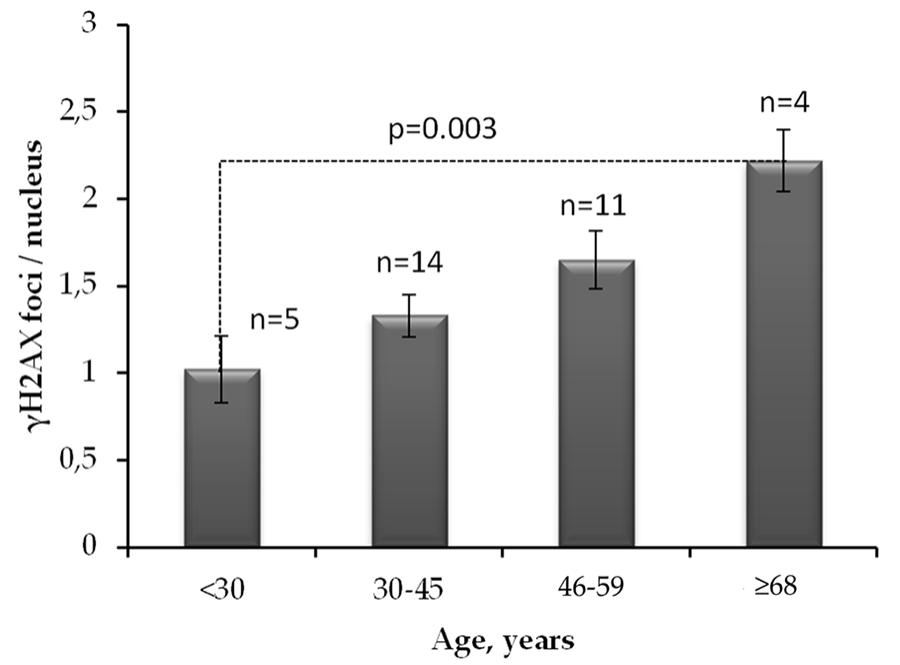
**The number of γH2AX foci in quiescent skin fibroblasts of donors of different age groups.** The data presented as Mean±SE.

The average number of γH2AX foci in quiescent fibroblasts of 68-year- and older donors was about two times as higher as the one in the cells of up to 30-year-old donors (Fig. 3)

### γH2AX foci number increases with proliferation activity rise

**Figure 4:**
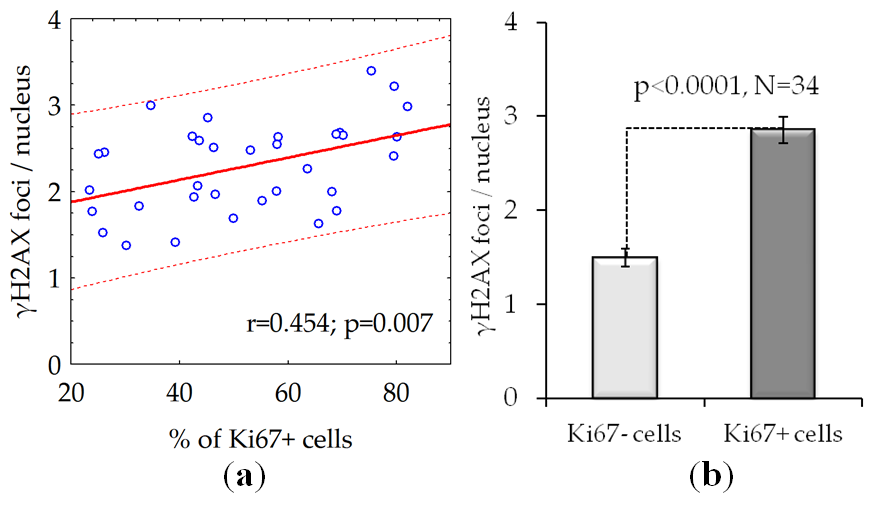
The number of γH2AX foci is dependent on the proliferative activity of cells: (**a**) The results of the correlation analysis; (**b**) The number of γH2AX foci in proliferating (Ki67 +) and quiescent (Ki67-) cells. The data presented as Mean±SE.

We found a statistically significant positive correlation between the fraction of proliferating cells and the number of γH2AX foci in fibroblast cultures from 34 donors (Fig. 4a). On average, the number of γH2AX foci in proliferating cells is about twice as many compared with the quiescent cells (Fig. 4b).

### The impact of senescence (SA β-gal+ cells) on γH2AX foci number in proliferating and quiescent fibroblasts

**Figure 5:**
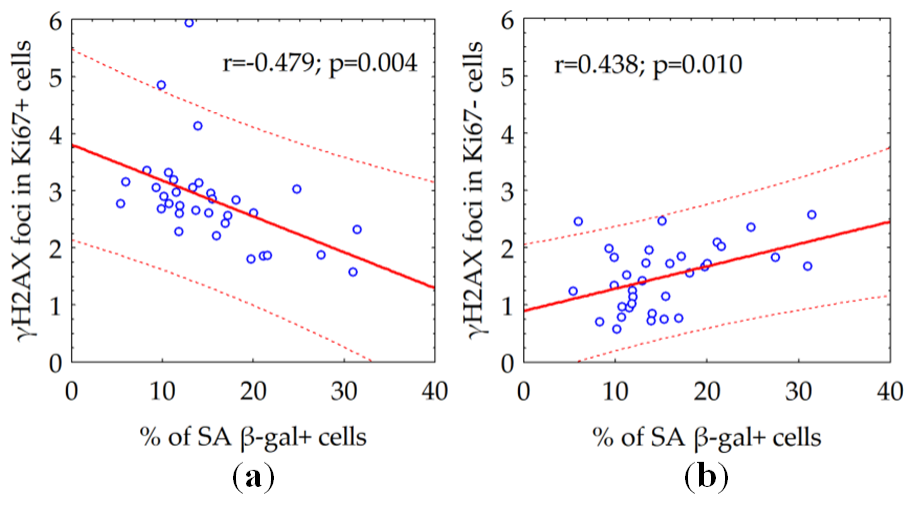
The correlation between the number of γH2AX foci in proliferating (**a**) or quiescent (**b**) cells and the fraction of senescent SA β-gal + cells.

Within a population of proliferating cells, we observed a declining the number of γH2AX foci relative to increased proportion of senescent SA β-gal + cells (Fig. 5a). Conversely, augmenting the number of γH2AX foci proportionally to the percentage of senescent (SA β-gal +) cells in the population of quiescent cells (Fig. 5b).

## DISCUSSION

In the present study for the first time we attempted the systematic correlation analysis of possible impact of donor age on clonogenicity, proliferative potential, and spontaneous γH2AX foci in the fractions of proliferating (Ki67 +), quiescent ((Ki67 -) and senescent (SA β-gal +) human skin fibroblasts isolated from 34 donors of different age. This allows a comparative assessment of donor's cell capacity to proliferate and their use as a valuable source for the development of optimal individual skin care protocols.

Cells isolated from biopsy material from different patients had varying ability for colony formation [26]. Here we showed no correlation between the parameters of clonogenicity and proliferative potential of fibroblasts, determined by the distribution of colony types, and the age of the donor. Our study corroborated previous data obtained by other researchers regarding the replicative lifespan of human cells [27].

Analysis of γH2AX as a biological marker of double-stranded DNA breaks has wide application in various biological studies, including for example, radiation bio-dosimetry [28], assessment of individual radio sensitivity [29], testing of medicinal and chemotherapeutic drugs [30–32], assessment of the effects of genotoxic environmental agents [33], studying the mechanisms of aging [34], etc. The processes of induction and repair of DSBs in proliferating and quiescent cells are significantly different [13]. About 11% of the human fibroblast nuclei contains spontaneous (constitutive) γH2AX foci. Among them, 9.2% represents proliferating cells carrying from 1 to 69 γH2AX foci / nucleus and 1.5% consist of non-proliferating cells containing from 1 to 4 γH2AX foci / nucleus [35]. For the first time, we demonstrated here positive correlation between donor age and the number of γH2AX foci accumulating in the fraction of quiescent (Ki 67-) cells (Fig.2c) in contrast to fraction of proliferating cells where the age-dependence was not observed (Fig.2b). Inasmuch as two times higher the constitutive level of γH2AX expression observed in live quiescent cells of the 68-year and older donors in comparison with up to 30-year-old ones (Fig.3), this likely indicates the age-dependent increase of the DNA damage by endogenous oxidants generated during respiration of this fraction of cells. Interesting, an average number of spontaneous γH2AX foci / nucleus increases proportionally to the growth of the overall percentage of proliferating (Ki67+) cells reaching two times as higher numbers comparing to quiescent (Ki67-) cells as demonstrated in our study (Fig.4a,b).

Next, we investigated whether the number of constitutive γH2AX foci associates with overall degree of senescence of human fibroblast cultures. We found the growth of the percentage of senescent (SA β-gal +) cells had two different effects depending on proliferative activity of the cells. Within a population of proliferating (Ki67+) cells, degree of senescence correlated with a relative declining (Fig. 5a) of constitutive γH2AX foci number, whereas in the population of quiescent (Ki67-) cells (Fig. 5b), it was proportional to augmenting the number of the γH2AX foci. One of the main sources of DSBs in proliferating cells is the collapse of replicative forks in the S phase of the cell cycle [36]. Therefore, the number of metabolic γH2AX foci in proliferating cells reflects the proliferative activity of cells. Thus, a negative correlation between the number of constitutive (metabolic) γH2AX foci and the fraction of senescent cells among proliferating cells might have a tempting logical explanation - the higher the proliferative activity, the lower the proportion of senescent cells among the fibroblast population and vice versa. The only exact mechanism of homologous recombination [36,37] repairs the resulting DSBs by ATR kinase mediating histone H2AX phosphorylation [38,39]. DSBs formed during DNA damage by various genotoxic factors, including free radicals formed during cellular respiration, have a completely different meaning for the fate of the cell and its descendants. These spontaneous (constitutive) DSBs are indicated by ATM kinase mediating H2AX phosphorylation [40]. Reparation of such DSBs occurs predominantly (up to 80%) using a quick but incorrect mechanism of non-homologous end joining with the frequent formation of micro-deletions and chromosomal rearrangements [41,42]. The accumulation of microstructural chromosomal abnormalities resulting from incorrect repair of DSBs seems to play a leading role in the process of cellular senescence [10,16,43]. DSBs formed in telomeric sequences played a particularly important role in the start of the cellular senescence. Since the repair of DSBs in telomeric sequences is difficult, they can accumulate over time as evidenced by the long presence of the γH2AX foci in telomeric DNA [44,45]. This process is related to cellular aging and the accumulation of γH2AX foci in senescent cells [45,46]. The 53BP1 foci and telomere-associated foci, the markers of DNA damage, accumulate with age of human donors [47]. Boosting ATM activity extends lifespan in a mouse model of progeria, thus emphasizing the relevance of the accumulation of γH2AX foci to alleviating aging [48]. The frequency of γH2AX foci-containing cells significantly increases with age in different tissues, including lung, spleen, dermis, liver and gut epithelium of mice [49]. Although accumulated DSBs in non-telomeric sequences also can play some role in senescence, our data might indicate the accumulation of unrepaired DSBs in telomeric sequences, driven by ongoing oxidative DNA damage, is, at least partly, responsible for the senescence of quiescent human fibroblasts. Moreover, present data on a statistically significant (p = 0.001) correlation between the age of the donor and the number of constitutive γH2AX foci in quiescent cells, could point out the continuing DNA-damage response due in the maintenance of the senescent cellular state.

From the point of further understanding of anti-aging cellular mechanisms, the present study supports the view that the aging of cells accompanied by the DSB accumulation. Indeed, it is the accumulation of DSBs, and the reduced DNA repair efficiency plays one of the critical roles in cellular aging. Accordingly, our research can serve as the blueprint for drug development (anti-aging cellular mechanisms) which should aim at preventing these adverse effects and demonstrating acceptable efficacy in extending life expectancy.

## MATERIALS AND METHODS

### Fibroblast isolation and culturing

By the study beginning, all the donors were medically examined and diagnosed as ‘‘healthy’’ by a dermatologist, physician, endocrinologist, and clinical immunologist. All the donors were non-smokers, did not live on the rural territories, and had no previous exposure to harmful environmental conditions. All human donors provided written consent for this study. The Russian Healthcare Regulation Authority and the Ethics Committee and Academic Council of the Central Research Institute of Dental and Maxillofacial Surgery (#4/276) granted ethics approval for the study. A skin biopsy was obtained with a disposable punch from behind the ear auricle under local infiltrative anesthesia with the 2% lidocaine solution. The isolated skin fragment (appx. 4 mm^3^) immediately transferred to the labeled sterile container with shipment medium (DMEM/F12). After that the biomaterial was transferred to a Petri dish under laboratory sterile conditions and washed three times with Hank’s solution supplemented with antibiotic (gentamycin) in Gibco® Versene solution (0.2 g EDTA(Na_4_) per liter of Phosphate Buffered Saline (PBS), pH 7.4) manufactured by Thermo Fisher Scientific, USA. The tissue homogenate was prepared by sequential dispersing with a sterile scalpel, the addition of the disaggregating 0.1% collagenase solution, and the incubation of disaggregating solution at 37° C for 1-1.5 hours.

After the incubation, vigorously mixing through 10-ml pipette used to make the tissue suspension after that centrifuged at 300 g for 10 minutes. Discarding the supernatant, and the pellet was resuspended again in the tissue culture medium (DMEM/F12, 1:1) containing 10% defined bovine fetal serum (FBS) (HyClone, USA) and 40ug/ml of gentamicin (TCM). The resulting suspension was transferred into a culture vial and incubated under the 5% CO_2_ atmosphere at 37° C in CO_2_-incubator with the exchange of the culture medium every 3-4 days.

At 70% of confluency, the cells were washed with Gibco® Versene solution and detached from the surface of a culture flask by use of Gibco® Versene solution and 0.25% trypsin at 37°C for 3-5 minutes. The culture medium diluted the cell suspension and explanted into a broader cultural flask for subsequent cultivation.

### Immunocytochemical analysis of γH2AX foci and Ki67 +/- cells

The cells seeded at the density of 5 × 10^3^ cells/cm^2^ in 0,5 mL of culture medium onto coverslips placed inside 35 mm Petri dishes (Corning, USA) for immunocytochemical analysis. To improve adhesion of cells additional volume of culture medium (1.5 mL) was added into Petri dishes 15 minutes after seeding. Cells seeded on coverslips were incubated at 37°C and 5% CO_2_ for at 48 h before fixation.

Cells were fixed on coverslips in 4% paraformaldehyde in PBS (pH 7.4) for 20 min at room temperature followed by two rinses in PBS and permeabilization in 0.3% Triton-X100 (in PBS, pH 7.4) supplemented with 2% bovine serum albumin (BSA) to block non-specific antibody binding. Cells were then incubated for 1 hour at room temperature with primary rabbit monoclonal antibody against γH2AX (dilution 1:200, clone EP854(2)Y, Merck-Millipore, USA) and primary mouse monoclonal antibody against Ki67 protein (dilution 1:400, clone Ki-S5, Merck-Millipore, USA) which were diluted in PBS with 1% BSA. After several rinses with PBS cells were incubated for 1 hour with secondary antibodies IgG (H+L) goat anti-mouse (Alexa Fluor 488 conjugated, dilution 1:600; Merck-Millipore, USA) and goat anti-rabbit (rhodamine conjugated, dilution 1:400; Merck-Millipore, USA) diluted in PBS (pH 7.4) with 1% BSA. Coverslips were then rinsed several times with PBS and mounted on microscope slides with ProLong Gold medium (Life Technologies, USA) with DAPI for DNA counter-staining. Cells viewed and imaged using Nikon Eclipse Ni-U microscope (Nikon, Japan) equipped with a high definition camera ProgRes MFcool (Jenoptik AG, Germany). Filter sets used were UV-2E/C (340–380 nm excitation and 435–485 nm emission), B-2E/C (465–495 nm excitation and 515–555 nm emission) and Y-2E/C (540–580 nm excitation and 600–660 nm emission). We imaged 300-400 cells for each data point and counted Foci by manual scoring.

### Analysis of β-galactosidase positive cells

To quantify the proportion of β-galactosidase positive cells the commercial kit “Cellular Senescence Assay» (EMD Millipore, USA, Catalog Number: KAA002) was used. The cells were stained according to supplemented manufacturer protocol with the following modification: at the final РВS washing step, the cell nuclei were stained with 1 μg/ml Hoechst 33342 (Molecular Probes, USA). Such modification significantly improves the quality of counting of β-galactosidase negative cells [25]. The stained cells were visualized using Excitation/Emission Interference Filters (СKX-U: 340-380nm/435-485 nm) on the inverted fluorescent microscope Оlympus СКХ 41 SF (Оlympus, Japan) equipped with Infinity 3-1 (Lumenera Copr., Canada) CCD camera and 20X objective.

### Clonogenic analysis

At second cell passage, the cell monolayer was washed three times with Gibco® Versene solution and trypsinized at 37°C, 5% CO_2_ for 10 min. The homogenate was centrifuged at 300 g for 10 minutes. The supernatant fluid discarded, the cells were resuspended in Hank’s solution, and counted in Gorjaev’s chamber.

The cells were diluted by TCM to obtain final concentration 100 cells/ml. The cell suspension split into three 100 mm Petri dishes to obtain a clonal inoculum density of 1.5 cells/cm^2^.

The Petri dishes were incubated in a CO_2_-incubator at the saturated humidity conditions at 37°C in the 5% CO_2_ atmosphere for 14 days. Thereafter, the culture dishes with the pre-formed colonies washed three times with PBS (рH 7.4 and fixed with 70% alcohol for 15 minutes at room temperature. Triple washing with distilled water used to remove alcohol residuals, and the colonies were stained with a KaryoMAX® Giemsa Stain Stock Solution produced by Gibco, USA, for 20 minutes at 37°C. Excessive stain thoroughly washed out from the dishes with the stained colonies and the dishes dried at room temperature for 5-7 hours.

After that, the analysis of the colonies was carried out. The colony forming effectiveness of the fibroblasts (ECO-f) was determined according to Fridenshtein’s equation for stromal progenitor cells: ECO-f = a ratio between the number of pre-formed colonies and the number of explanted cells multiplied by 100% [50,51]. The only counted colonies include more than 20 cells of the total number of explanted cells. The clones of fibroblasts which consisting of fewer cells were not considered as colonies and, accordingly, were not included in the count.

For calculation of fibroblast proliferative potential (РР) the colonies were ranked in three groups: dense, diffuse and mixed colonies and using the following formula: PP = [1(DC) + 2(MC) + 3(CC)] / 100%, where DC, MC and CC are percentages of diffuse, mixed and compact colonies [25].

### Statistical analysis

Statistical and mathematical analyses of the data were conducted using the Statistica 8.0 software (StatSoft). The Pearson correlation coefficient, also referred to as Pearson's r, used as a measure of the linear correlation between two variables X and Y in our experimental data analysis. Statistical significance was tested using the Student t-test at p < 0.05.
